# Emerging Bacterial Resistance and Genotoxicity of Water-Soluble Fractions of Agricultural Soils from the Semiarid Region of Brazil Affected by the Continuous Use of Glyphosate

**DOI:** 10.1007/s00128-026-04230-1

**Published:** 2026-04-06

**Authors:** Karolayne Silva Souza, Amanda Alves de Araujo, Milena Roberta Freire da Silva, Kátia Cilene da Silva Felix, Kaline Catiely Campos Silva, Ricardo Marques Nogueira Filho, Lívia Caroline Alexandre de Araujo, Amanda Vieira de Barros, Fabricio Motteran, Elvis Joacir De França, Claudia Rohde, Maria Betânia Melo de Oliveira

**Affiliations:** 1 Universidade Federal de Pernambuco, Recife, Pernambuco Brazil; 2 Programa de Pós-Graduação em Biologia Celular e Molecular Aplicada, Universidade Federal de Pernambuco, Vitória de Santo Antão, Recife, Pernambuco Brazil; 3 Centro Acadêmico de Vitória, Universidade Federal de Pernambuco, Vitória de Santo Antão, Pernambuco Brazil; 4 Centro Universitário do Rio São Francisco, Paulo Afonso, Bahia Brazil; 5 Universidade do Estado da Bahia, Paulo Afonso, Bahia Brazil; 6 Centro Regional de Ciências Nucleares do Nordeste, Recife, Pernambuco Brazil

**Keywords:** Pesticides, Microbiota, Petrolândia, Comet assay, *Drosophila melanogaster*

## Abstract

The use of herbicides in modern agriculture has been the subject of ongoing debate due to their negative impacts on the environment and human health. The aim of this study was to assess the bacterial, physicochemical and genotoxicity parameters of three soil samples of public farming settlements in Petrolândia, Brazil. The bacterial resistance patterns were examined after soil exposures to a known concentration of glyphosate (2.5%). Genotoxicity was assessed through the *Drosophila melanogaster* Comet assay, exposed to water-soluble fraction (WSF) soil samples, followed by the quantification of seven chemical elements. The results indicated that, although physicochemical parameters remained within acceptable limits, there was a significant presence of resistant bacteria, particularly *Stenotrophomonas maltophilia*, *Bacillus cereus* and *Enterobacter cloacae*. Comet assay results (Damage Index and Damage Frequency) presented a significant level of genotoxicity in two of the three WSFs. Clustering analysis associated the genetic damage to the presence of chrome and zinc heavy metals. The predominance of multidrug-resistant bacteria, combined with levels of genotoxicity, suggests that these soils exposed to glyphosate contamination represents a risk to the sustainability of Brazilian semiarid agriculture.

**Supplementary Information:**

The online version contains supplementary material available at 10.1007/s00128-026-04230-1.

## Introduction

Brazil is one of the largest consumers of pesticides globally, with particularly intensive use on crops such as soybeans, maize and sugarcane. Glyphosate [N-(phosphonomethyl)glycine] is the active ingredient in Roundup®, the most widely used herbicide worldwide. It is notable for its effectiveness in controlling a broad spectrum of weeds, and for its role in maximizing agricultural productivity to meet the growing demand for food (Clapp 2021; Lima et al. 2021; Muller et al. 2021; Lopes-Ferreira et al. 2022). However, its widespread use raises serious concerns regarding the adverse effects of continued application on soil and associated ecosystems (Meftaul et al. 2020; Lopes-Ferreira et al. 2022; Qiu et al. 2022; Zhang et al. 2024).

Bacterial resistance stands among the most concerning consequences of the indiscriminate use of glyphosate, as resistant bacteria can emerge and proliferate in environments where the herbicide is regularly applied, posing a threat not only to agriculture, but also to human and animal health (Singh et al. 2024; Zhang et al. 2024; Li et al. 2025). According to Puigbò et al. (2022) a healthy microbiota contains diverse species that are either sensitive or resistant to glyphosate-based products due to their action on the target site of the 5-enolpyruvylshikimate-3-phosphate synthase (EPSPS) enzyme (Leino et al. 2021). By analyzing a dataset of the 732 bacterial genomes, Puigbò et al. (2022) demonstrated that over half of the human microbiome is intrinsically sensitive to glyphosate, which may promote the spread of resistant, fast-evolving bacteria and cause ecological impacts in exposed organisms (Gandhi et al. 2021; Zhang et al. 2024).

As the pesticide database provided by the Brazilian Institute of Environment and Renewable Natural Resources (IBAMA), in 2010 and 2020 Brazil sold 157,512 and 329,697 tons of herbicide active ingredients, respectively, representing a 128.1% increase in sales over 11 years (Procópio et al. 2024). Glyphosate is primarily used due to its low cost for weed control, rapid absorption by plants, and the widespread misconception of its low toxicity to the environment and living organisms (Rivas-Garcia et al. 2022). However, Dias et al. (2023) demonstrated the environmental impact of glyphosate use in Brazilian soybean cultivation and its externality effects in downstream water sources in cultivated areas. The authors identified glyphosate as the principal contaminant affecting populations indirectly exposed, rather than only those directly handling the herbicide. Their study detected negative effects on birth outcomes in exposed populations and addressed the empirical challenges typical of estimating the environmental toxicology impacts of pesticide use.

According to Oliveira et al. (2024) very few studies present data regarding soil fragility in the Brazilian semiarid environments, the most populous semiarid region in the world and home to more than 54 million inhabitants (IBGE 2023). In Northeastern Brazil, the Icó-Mandantes and Apolônio Sales irrigation projects, located in the municipality of Petrolândia, in the state of Pernambuco, represent significant areas of agricultural production. In 2023, the Icó-Mandantes project produced 38,014 tons of family agricultural output, while Apolônio Sales generated 23,628 tons (CODEVASF 2024). Based on local information, both projects rely heavily on glyphosate for weed control, making them ideal semiarid areas for studying the environmental impacts and soil health consequences associated with the prolonged use of glyphosate.

Soil is a highly complex ecosystem that provides essential resources and biological processes that are fundamental to support interactions among soil flora and fauna of invertebrates and vertebrates (Ruess et al. 2025). However, the introduction of anthropogenic substances can disrupt these interactions, affecting the abundance, diversity and distribution of specific organisms. Numerous investigations have highlighted the health risks of glyphosate and glyphosate-based herbicides to higher animals, including cytotoxicity, genotoxicity, oxidative stress, disruption of estrogen pathways, and pro-inflammatory responses (Li et al. 2025). Therefore, it is crucial to obtain a better understanding of the thresholds at which soil contaminants pose risks and cause immediate damage to DNA (genotoxic effect). One of the most widely applied methodologies in genetic ecotoxicology is the Comet assay, which has revolutionized the field and has been applied to invertebrate model-organisms, such *Drosophila melanogaster* (Diptera, Drosophilidae) (Santana et al. 2018; Nascimento-Silva et al. 2024; Cipriano et al. 2026).

Given this scenario, this study has aimed to assess bacterial resistance to glyphosate, soil quality parameters, the presence of chemical contaminants in the water-solution fraction (WSF) of soil samples from glyphosate-treated areas, and genotoxicity. As WSF contains a wide range of dissolved chemical elements, which are bound to nanoparticles and colloids, it has the capability to facilitate the transport of nutrients, as well as heavy metals, pesticides and other contaminants (Weihrauch and Opp 2018). 2019). The mobile chemical elements dissolved in this fraction, as well as the components bound to nanoparticles and colloids, reflecting the soil properties when partitioned into the water-soluble phase (Panova et al*.* 2020).These fractions are widely distributed in the environment, forming deposits with varying concentrations, some playing essential roles in organic metabolism, and others presenting toxic potential in physiological processes (Alengebawy et al. 2021). Depending on tissue concentration, exposure to metals such as Cd and Zn can induce mitotic inhibition, mutagenicity, and Comet tails (DNA breaks) as demonstrated by Jayawardena et al. (2021) in the erythrocytes of *Euphlyctis hexadactylus* and bioassays with *Allium cepa*. The combined assessment of bacteriological, chemical, and genotoxic parameters aims to understand the impacts of the continued use of glyphosate in family agricultural production, to provide new insights and to enhance more sustainable agricultural practices for the Brazilian semiarid region.

## Material and Methods

### Soil Samples and Analysis

Soil samples were collected in duplicate, stored in thermal boxes, and named P1 (− 8.8286, − 38.3000), P2 (− 8.9070, − 38.4143) and P3 (− 8.9501, − 38.2714). P1 and P2 were located in the Icó-Mandantes irrigation project and P3 in the Apolônio Sales irrigation project, in the municipality of Petrolândia, in the state of Pernambuco, Brazil, two prominent examples of agricultural irrigation projects that rely heavily on the use of glyphosate herbicide for weed control. These locations were strategically chosen to represent the diverse environmental and management conditions prevailing in these areas. Due to technical reasons and difficulties in its detection (review in Singh et al. 2020), the quantification of glyphosate in soil samples was not performed in this study. The estimation of its continuous use was based on information from family farmers of the studied areas. As glyphosate is degraded by soil microflora, both under aerobic and anaerobic conditions (Giesy et al. 2000), the metabolic pathways of this process generate other compounds, such as CO_2_ and aminomethylphosphonic acid (AMPA). According to Mérey et al. (2016), AMPA continues to be degraded in the soil, and may represent between 13 and 50% of glyphosate-derived residues at the site, which poses challenges for its chemical characterization. To preserve the microbiological integrity of samples, the soil was stored at 4 °C until microbiological tests could be performed, enabling an integrated assessment of soil quality, bacterial resistance and genotoxic effects. Soil density, particle size composition, pH, available phosphorus (P), and exchangeable cations (Ca, Mg, Na, K) were determined following the methodologies described in the *Manual of Soil Analysis Methods* (EMBRAPA 2017) (Table S1, Supplementary Material).

### Biochemical Bacterial Identification and Susceptibility Profile

Homogenized soil samples (20 g) were transferred into glass flasks containing a disaggregation solution, prepared with sodium pyrophosphate (0.18 g), Tween 80 (0.18 mL), NaCl (1.53 g), and distilled water (174.6 mL). To this solution 5.4 mL of pre-filtered commercial glyphosate (Nufosate—Sumitomo Chemical Brasil Ind. Química SA) was added, resulting in a final glyphosate concentration of approximately 2.5%. Glass beads were included in the mixture to facilitate the disaggregation and the flasks were placed in a shaker incubator at 150 rpm for 30 min at 37 °C. After incubation, an aliquot from each flask was collected to initiate serial dilutions, considering the initial sample as the first dilution (10^-1), and subsequent dilutions (10^-2 to 10^-5) were performed in tubes of sterile saline solution (0.85%).

For bacterial isolation, 50 µL of each dilution was spread onto Eosin Methylene Blue (EMB) agar, 5% blood agar, and nutrient agar using a Drigalski spatula, then incubated at 37 °C and monitored at two intervals: 24 h (Group A) and 48 h (Group B). After the incubation period, distinct bacterial colonies were isolated by transferring portions of each colony to tubes containing Nutrient agar enriched with glyphosate, where they were stored for further analysis and characterization. Bacterial colonies were identified based on Gram staining and subsequently characterized using the MicroScan® autoSCAN 4® system (Dade Behring—West Sacramento, California, USA). For Gram-positive isolates, the Pos Combo 21 panel was employed, and for Gram-negative isolates, the Neg Combo 32 panel was used.

Susceptibility determination was performed by determining the Minimum Inhibitory Concentration (MIC), with interpretations conducted according to the standards of the Clinical and Laboratory Standards Institute (Gaur et al. 2023), classified as Sensitive (S), Intermediate Resistance (I), or Resistant (R) following the Clinical and Laboratory Standards Institute (CLSI) guidelines. Based on the acquired resistance profiles, bacterial isolates were then categorized into the following resistance patterns: Multidrug Resistant (MDR), Extensively Drug Resistant (XDR), Pan-drug Resistant (PDR), and Non-multidrug Resistant (N-MDR).

Taxonomic identification of bacterial isolates was confirmed using matrix-assisted laser desorption/ionization time-of-flight mass spectrometry (MALDI-TOF MS). Cultures grown in brain heart infusion (BHI) medium for 24 h were resuspended in deionized water, and proteins were extracted following the protocol described by Starostin et al. (2015). For mass spectrum analysis, 1 μL of each protein extract was deposited onto a 96-spot MSP plate (Bruker Daltonics, Billerica, MA, USA), and air-dried at room temperature. Subsequently, the alpha-cyano-4-hydroxycinnamic acid matrix prepared in 50% acetonitrile (v/v) and 0.3% trifluoroacetic acid (v/v) was applied to the plate to allow crystallization. Mass spectra were acquired in positive linear mode using an acceleration voltage of 20 kV and a mass detection range of m/z of 2,000–20,000, using Flex Control 3.0, and a MALDI-TOF Autoflex III spectrometer (Bruker Daltonics). Validation for the target organisms was performed following the log-score criteria established by the manufacturer of the Bruker Biotyper MALDI-TOF MS system. According to international standards, score values > 2.00 were adopted for reliable identification at the species level, and between 1.70 and 1.99 for the genus level.

### Chemical Analysis of the Water-Soluble Fraction (WSF) of Soil

The water-soluble fraction (WSF) represents the portion of soil components that dissolve in water (hydrosoluble fraction), forming an aqueous solution suitable for chemical and biological analyses. The WSF of *in natura* sample soils from P1, P2 and P3 were prepared following a water pH protocol, described by Sahuquillo (1999) with modifications proposed by Silva (2025). Initially, 500 g of each soil sample was weighed and oven-dried until constant weight was achieved and water was eliminated. Approximately 10 g of each dried soil sample was then ground into a fine powder using a mortar and pestle. For each soil sample (P1, P2 and P3), 1 g of the powder was transferred into a Falcon tube, followed by the addition of 40 mL of Milli-Q water. The suspensions were then mechanically agitated for 16 h, after which, the supernatant was separated to obtain the WSF. This process was performed in triplicate for each soil sample, generating three replicates for the same chemical and genotoxicity testing, using the Comet assay.

Chemical trace-level elemental analysis of WSFs was performed using graphite furnace atomic absorption spectrometry (GFAAS), a technique well-suited for small sample volumes and capable of achieving very low detection limits. For the preparation of calibration standards, thirteen sterile 15 mL Falcon tubes with caps (previously weighed and numbered) were used. Standard concentrations of each element (Merck) were added to the tubes numbered from 1 to 8 as follows: 1 mL for aluminum (Al) and 0.100 mL for cadmium (Cd), cobalt (Co), chromium (Cr), manganese (Mn), and lead (Pb). All tubes were filled to a final volume of 15 mL with 5% nitric acid (prepared from 65% analytical-grade nitric acid diluted in Milli-Q water). After assembling the calibration standard for each element, in tube 9 (sterile and pre-weighed), the multielement solution was prepared by adding the standard concentration of each element: 0.8 mL of the Al solution; 0.1 mL of Cd and 1.2 mL of the solution of the other elements mentioned above. After each addition, the weight was checked. From the multi-element solution in tube 9, a series of dilutions was prepared in tubes 10 to 13 to establish the calibration curve and verification standard. Specifically, 1.4 mL, 1.0 mL, 0.5 mL, and 0.7 mL of the multielement solution were added to tubes 10, 11, 12, and 13, respectively. Tubes 10 to 12 were used to construct the GRAAS calibration curve, while tube 13 served as the verification standard during sample analysis.

Due to the high levels of Zinc (Zn) detected in the preliminary GFAAS analysis of WSF, flame atomic absorption spectrometry (FAAS) was employed for more accurate quantification. In this technique, the sample is aspirated into a flame, where zinc is vaporized and atomized. A beam of light is then passed through the flame, and the amount of light absorbed by the zinc atoms is measured. This measured absorption is directly proportional to the zinc concentration in the sample. Standard solutions and analytical blanks were prepared using the VARIAN AAS 220 FS spectrometer. Instrumental parameters and calibration curves were established using Merck-certified standard solutions with known concentrations of Zn, optimized by adjusting the hollow cathode lamp current and the acetylene-air gas flow rates.

### Genotoxic Analysis of the WSF

The WSFs were assessed for their potential to induce genetic damage in the DNA of somatic cells of third instar larvae of *Drosophila melanogaster* Oregon-R strain, a previouly validated strain (Verçosa et al. 2017). The Comet assay is a method that allows the detection of single- and double-strand DNA breaks, alkali labile sites (apurinic/apyrimidinic sites), DNA cross-links, base and base-pair damage, and apoptotic nuclei in the exposed cells. The genotoxicity was assessed with liquid WSFs, which contain an unknown combination of chemicals (such as heavy metals) and also residues of herbicides, probably glyphosate.

### Sample Preparation and Hemolymph Extraction

The WSFs were incorporated into the culture medium, composed of dehydrated commercial mashed potatoes (Yoki SA), on which the 60 *D. melanogaster* 3rd instar larvae were allowed to feed for one day. Each experiment was performed in triplicate. The negative control group was composed of commercial mashed potatoes hydrated with distilled water. The positive control group was composed of commercial mashed potato hydrated with a compound with recognized mutagenic effect (Cyclophosphamide 2 mg/mL, diluted in distilled water), following previous descriptions (Santana et al. 2018; Nascimento-Silva et al. 2024).

After the feeding period in the WSF-supplemented medium, hemolymph cells of 60 *D. melanogaster* larvae per replicate were extracted together, on concave Kline glass plates containing 150 μL of anticoagulant solution (3 g NaOH, 14.89 g EDTA, 200 mL H_2_O, adjusted to pH 10). The cell suspension with hemocytes was collected in a 1.5 mL microtube, centrifuged twice at 3000 rpm for 3 min, homogenized in 100 μL of low-melting point agarose (0.5%) heated at 37 °C, and mounted on glass slides previously sanded and soaked in standard agarose (1.5%). All procedures were performed under red light in a dark room to minimize potential DNA damage caused by light exposure. The slides were covered with coverslips and placed at 4 °C to solidify the agarose. After the preparation of four slides per each replicate, the coverslips were removed and the glass slides with the biological material were immersed in lysis solution (NaCl 2.5 M, EDTA 100 mM, NaOH 1 M, Tris 1% pH 10, Triton X-100 1%, and DMSO 10%) at 4 °C for 72 h.

### Electrophoresis and DNA Damage Assessment

After lysis, electrophoresis was carried out for 20 min at 40 V and 300 mA in a 40 cm vat, followed by the neutralization and fixation stages (Nascimento-Silva et al. 2024). For the evaluation of DNA damage, nucleoids were stained with 50 μL of GelRed dye (Biotium), diluted 1:500 in distilled water, and examined under a Zeiss Axio Imager M2 fluorescence microscope at 400 × magnification using a 546 nm filter. One hundred cells were analyzed per each three replicates of P1, P2 and P3, and the nucleoids were classified into damage levels from 0 to 4, according to the visual inspection criteria (Collins et al. 2008; Verçosa et al. 2017). Damage level 0 corresponds to intact nucleoids; levels 1, 2, and 3 represent minimal, moderate and severe damage, respectively; and level 4 corresponds to maximum damage, characterized by long comet tails and minimum comet heads. After quantification of the damage, two measurements were calculated for each experimental replicate: Damage Index (DI) and Damage Frequency (DF%), according to the following equations, where *N1*, *N2*, *N3*, *N4* represent the total number of nucleoids with damage levels 1, 2, 3 and 4, respectively; *NT* represents the total number of nucleoids analyzed; and *N0*represents the total number of damage level *0*.

$$ {\text{DI = }}0.\left( {N0} \right) + {1}.\left( {N1} \right) + {2}.\left( {N2} \right) + {3}.\left( {N3} \right) + {4}.\left( {N4} \right) $$

$$ {\text{DF }}\left( \% \right) = \left[ {\left( {NT - N0} \right).{1}00} \right] \div NT $$

DI values can range from 0 (fully intact, i.e., 100 cells × 0) to 400 (maximum damage, i.e., 100 cells × 4) and DF% ranges from 0 to 100%.

### Clustering Analysis

The relationships between seven chemical variables (Al, Cd, Co, Cr, Mn, Pb and Zn) present in the WSF soil samples and the biological genetic damage observed after the Comet assay (Damage Index and Damage Frequency) were analyzed thought a cluster analysis. This method identifies patterns of similarity and dissimilarity within the dataset, allowing the grouping of variables with similar behavior (Araújo et al. 2024). The results were visualized through a dendrogram, which graphically represents the relationships between variables, highlighting data groupings with greater proximity.

### Statistical Analyses

Statistical analyses of the Comet assay were performed using analysis of variance (ANOVA) followed by the Bonferroni post hoc test for pairwise comparisons, using STATA 14.2 software, with a significance level of *P* ≤ 0.05. To investigate the relationship between the chemical elements present in the WSF and the genetic damage indicators (DI and DF%) obtained from the Comet assay, a multivariate cluster analysis was conducted using the City Block distance method (or Manhattan), which was calculated as the sum of absolute differences between variables, and Ward’s method was applied as the hierarchical amalgamation method. Cluster analyses were conducted using Statistica software, version 7.0 (StatSoft Inc., Tulsa, OK, USA).

## Results

The physicochemical analyses of soil samples are presented in Table 1, and were compared to the threshold values set by the Brazilian Agricultural Research Corporation (EMBRAPA 2017).

**Table 1:** Physicochemical results of soil samples P1, P2 and P3 and reference values from EMBRAPA (2017)

Parameter	P1	P2	P3	Reference values
Physical analysis				
Apparent density (g/cm^3^)	1.43	1.54	1.57	1.1–1.6
Particle density (g/cm^3^)	2.62	2.62	2.62	≈ 2.65
Coarse sand (%)	41	43	45	0.2–2 mm (particle diameter)
Fine sand (%)	47	47	47	0.05–0.2 mm (particle diameter)
Silt (%)	2	2	2	0.002–0.05 mm (particle diameter)
Clay (%)	10	8	6	< 0.002 mm (particle diameter)
Chemical analysis				
Available phosphorus (P) (mg/dm^3^)	92	43	29	≥ 20 for agricultural soils
pH	7.30	6.90	7.40	5.5–7.0
Calcium (Ca) (cmolc/dm^3^)	2.45	2.50	2.00	1.5–6
Magnesium (Mg) (cmolc/dm^3^)	0.75	1.20	0.85	0.5–2
Sodium (Na) (cmolc/dm^3^)	0.03	0.06	0.02	< 0.7
Potassium (K) (cmolc/dm^3^)	0.17	0.26	0.22	0.1–0.3
Aluminum (Al) (cmolc/dm^3^)	0.00	0.00	0.00	< 0.5

A total of 28 bacterial isolates were identified (10 isolates in P1, 10 in P2, and 8 in P3, listed in Table 2). Of these isolates, 15 (54%) were gram-positive bacteria (*Bacillus cereus, Bacillus megaterium, Staphylococcus siniae, Staphylococcus capitis, Clostridium difficile, Paenibacillus* sp*.,* and *Paenibacillus agaridevorans)* and 13 (46%) were gram-negative bacteria *(Enterobacter cloacae, Stenotrophomonas maltophilia, Pseudomonas nitroreducens, Shewanella frigidimarina, Klebsiella variicola, Burkholderia gladioli, Prevotella heparinolytica*, and *Rhizobium radiobacter*). The most prevalent genus was *Bacillus*, identified in nine out of 28 isolates across different collection points. *Bacillus cereus* species was the most frequently isolated.

**Table 2:** Classification of resistance profiles of bacterial isolates from P1, P2, and P3 soil samples (MDR, multidrug-resistant; N-MDR, non-multidrug-resistant

Points	Species	Antibiotics	Classification	Score MALDI-TOF MS
P1—Icó-Mandantes				
P1D1	*Bacillus cereus*	AMOX, AMP, CEF, OXA, PEN	N-MDR	2.12
P1D2A	*Bacillus megaterium*	CLIN	N-MDR	2.15
P1D2B	*Enterobacter cloacae*	AMOX, AMP	N-MDR	2.20
P1D2C	*Enterobacter cloacae*	AMOX, AMP	N-MDR	2.25
P1D2D	*Enterobacter cloacae*	AMOX, AMP	N-MDR	2.30
P1D3A	*Pseudomonas nitroreducens*	COLI	N-MDR	2.09
P1D3B	*Bacillus cereus*	AMOX, AMP, CEF, OXA, PEN	N-MDR	2.18
P1D3C	*Stenotrophomonas maltophilia*	AMI, CEFE, CIP, GENT, IMI, MER, PIP, TRO	MDR	2.28
P1D3D	*Enterobacter cloacae*	AMOX, AMP	N-MDR	2.14
P1D3E	*Stenotrophomonas maltophilia*	AMI, CEFE, CIP, IMI, MER, PIP, PIPE, TOB	MDR	2.32
P2—Icó-Mandantes				
P2D1A	*Bacillus cereus*	AMOX, AMP, CEFT, OXA, PEN	N-MDR	2.21
P2D2A	*Clostridium difficile*	AMOX, GENT, SIN	N-MDR	2.16
P2D2B	*Bacillus cereus*	AMOX, AMP, CEFT, OXA, PEN	N-MDR	2.11
P2D3A	*Shewanella frigidimarina*	AZT, COLI	N-MDR	2.08
P2D3B	*Staphylococcus siniae*	AMOX, AMP, CEFT, CLIN, OXA, PEN	MDR	2.19
P2D3C	*Klebsiella variicola*	AMP, PIP, FOS	MDR	2.27
P2D3D	*Brukholderia gladioli*	AMP, FOS	N-MDR	2.10
P2D3E	*Bacillus cereus*	AMOX, AMP, CEFT, OXA, PEN	N-MDR	2.13
P2D3F	*Stenotrophomonas maltophilia*	AMOX, CEFE, CEFO, CEFT, COLI	MDR	2.22
P2D3G	*Bacillus cereus*	AMOX, AMP, CEFT, OXA, PEN	N-MDR	2.29
P3—Apolônio Sales				
P3D1A	*Prevotella heparinolytica*	AZT, COLI, ERT, FOS	N-MDR	2.24
P3D2A	*Bacillus cereus*	AMOX, AMP, CEFT, OXA, PEN	N-MDR	2.17
P3D3A	*Rhizobium radiobacter*	AZT, FOS	N-MDR	2.23
P3D3B	*Staphylococcus capitis*	CLIN	N-MDR	2.09
P3D3C	*Paenibacillus sp*	–	N-MDR	2.20
P3D3D	*Bacillus cereus*	AMOX, AMP, CEFT, OXA, PEN	N-MDR	2.30
P3D3E	*Pichia accidentalis*	–	N-MDR	2.25
P3D3F	*Paenibacillus agaridevorans*	AMOX, AMP, CEFT, OXA, PEN	N-MDR	2.15

Antibodies: Amikacin (AMI); Amoxicillin (AMOX); Ampicillin (AMP); Aztreonam (AZT); Cefalotina (CEF); Cefepime (CEFE); Ceftriaxone (CEFT); Ciprofloxacin (CIP); Clindamycin (CLIN); Colistin (COLI); Ertapenem (ERT); Fosfomycin (FOS); Gentamicin (GENT); Imipenem (IMI); Meropenem (MER); Oxacillin (OXA); Penicillin (PEN); Piperacillin (PIP); Piperacillin/Tazobactam (PIPE); Synercid (Quinupristin/Dalfopristin) (SIN); Tobramycin (TOB); Trimethoprim-Sulfamethoxazole (TRO).

The bacterial resistance analysis, presented in soil samples supplemented with 2.5% glyphosate, revealed a diverse antibiotic resistance profile (Table 2). Five isolates (19%) were classified as multidrug-resistant (MDR), in P1 and P2 soil samples, and altmost 23 (81%) isolates were non-multidrug-resistant (N-MDR). Among the isolates, *S. maltophilia* demonstrated the highest resistance profile, displaying resistance to eight antibiotics (AMI, CEFE, CIP, GENT, IMI, MER, PIP, TRO). Other species that presented significant resistance was *S. siniae*, identified at point P2D3B, which was resistant to four different antibiotic classes (AMOX, AMP, CEFT, CLIN, OXA, and PEN), and *K. variicola*, found at point P2D3C, resistant to two classes (AMP, PIP, and FOS).

The genotoxicity results of the Comet assay (Table 3) corroborate the bacterial resistance patterns observed, with higher levels of DI and DF% in cells exposed to P1 and P2, compared to P3. According to the Bonferroni statistical analyses (Table S2 of Supplementary Material), no significant differences in DI were observed between P1 and P2 (P = 0.109) localized in the Icó-Mandantes project, or between P in the Apolônio Salas project and the negative control group (P = 0.109). The same results were obtained for DF%, with no significant differences observed between of P1 and P2 (P = 0.292), or between P3 and negative control (P = 1.000).

**Table 3:** Mean number of damage index (DI) and mean damage frequency (DF%) and Standard deviation (Sd) (n = 3 replicates) in hemocytes of *Drosophila melanogaster* larvae exposed to soil samples (P1, P2 and P3), as well as the negative control (CO–) (distilled water) and positive control (CO +) (Cyclophosphamide 2 mg/mL)

Controls	Damage levels (media ± Sd)					DI ± Sd	DF% ± Sd
	0	1	2	3	4		
CO-	82.67 ± 2.08	0.67 ± 1.15	12.33 ± 0.58	2.67 ± 1.53	1.67 ± 2.08	40.00 ± 8.19	17.33 ± 2.08
CO +	62.00 ± 1.00	7.33 ± 1.15	12.00 ± 1.00	5.33 ± 2.52	13.33 ± 2.31	*100.67 ± 4.04	*38.00 ± 1.00
Soil samples							
P1	67.67 ± 2.08	1.33 ± 0.58	2.33 ± 1.53	7.33 ± 0.58	21.33 ± 2.31	*113.33 ± 7.51	*32.33 ± 2.08
P2	62.67 ± 0.58	1.00 ± 1.00	1.67 ± 1.53	7.33 ± 3.21	27.33 ± 3.06	*135.67 ± 5.69	*37.33 ± 0.58
P3	81.00 ± 4.36	2.33 ± 1.15	2.00 ± 2.65	2.67 ± 1.53	12.0 ± 2.00	62.33 ± 14.57	19.00 ± 4.36

*Means significant differences (*P* ≤ 0.05) comparing to negative control group

### Chemical Analysis and Clustering with Genetic Damage

**Fig. 1:**
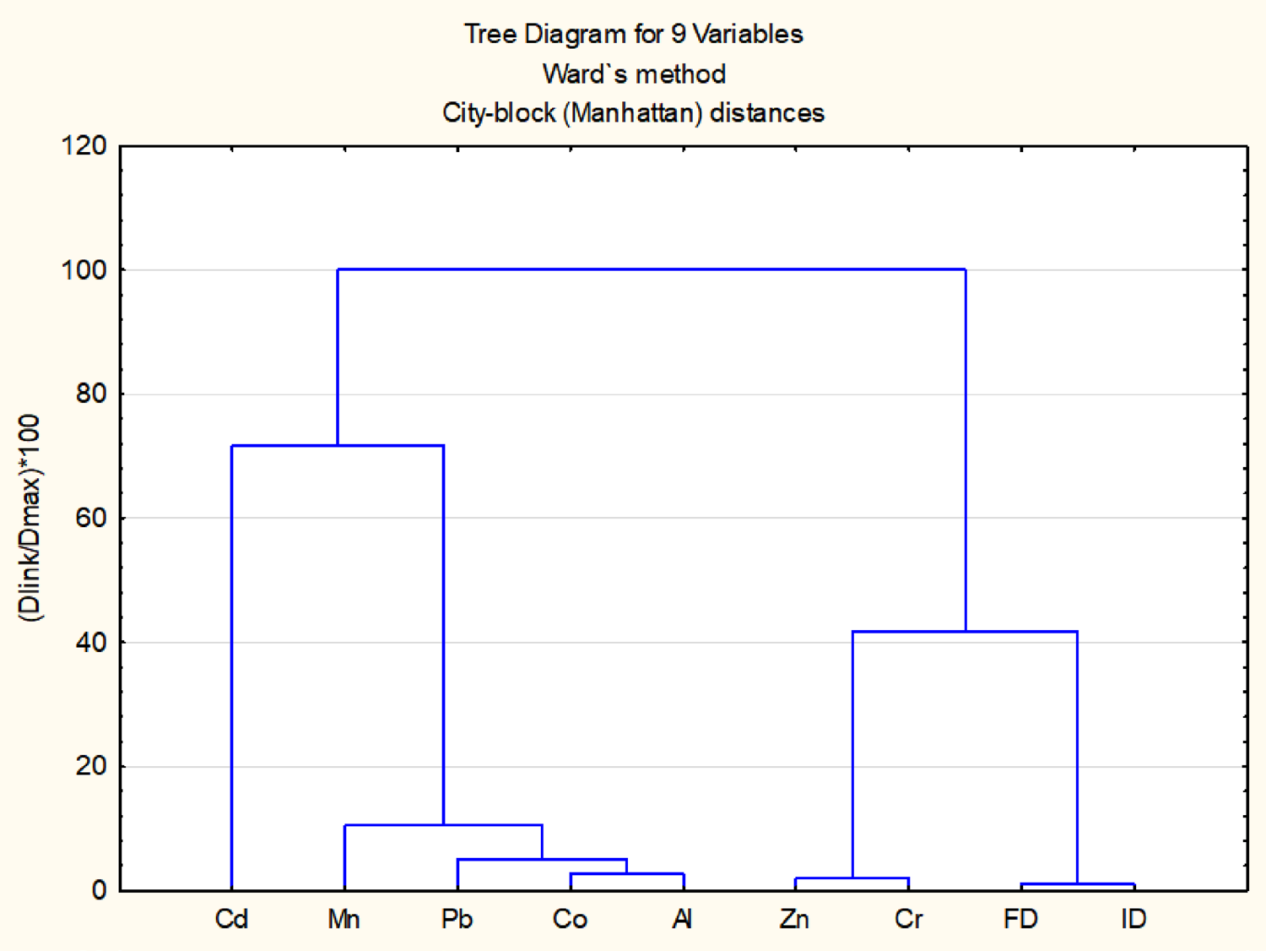
Representation of the clustering relationship among the nine variables generated by cluster analysis, based on City Block (Manhattan) distance and Ward's method. Cd – Cadmium, Mn – Manganese, Pb – Lead, Co – Cobalt, Al – Aluminum, Zn – Zinc, Cr – Chromium, FD – Damage Frequency, ID – Damage Index

The quantification results of each chemical element (Cd, Mn, Pb, Co, Al, Zn and Cr) are presented in Table S3 of the Supplementary Material. The highest presence of some chemicals (µg/L) were observed in P1 (as Al, 53.72; Co, 2,62; Cr, 22.09; Mn, 33.95; Pb, 9,80 and Zn, 0.04), followed by P3 (Al, 32.62; Cd, 0.59; Co, 0.71; Cr, 10.02; Mn, 31,34; Pb, 4.52; Zn, 0.02) and P2 (Al, 30.77; Cr, 16.79; Mn, 29.97; Pb, 4.76; Zn, 0.03). The dendogram presented in Fig. 1 shows the attribute distances between each pair of sequentially merged classes, identifying patterns of similarity and dissimilarity within the seven chemical elements analyzed and the genotoxic dataset (Damage Index and Damage Frequency). The results presented through the dendrogram facilitated the interpretation of complex relationships among the quantified variables, and revealing strong clustering association of Zn and Cr with the clustering of DI and DF%.

## Discussion

The results of the physicochemical analyses revealed that the bulk density of the soil samples ranged from 1.43 to 1.57 g/cm^3^, which falls within the acceptable range for agricultural soils. Particle density was consistent across all samples, with a value of 2.62 g/cm^3^, aligning with the typical values for mineral soils. These parameters indicate that the soils possess good structural quality, with minimal compaction, thereby favoring root penetration and water infiltration.

Particle size distribution of the samples indicated that the soils are predominantly sandy. This texture can influence the soil’s capacity for water and nutrient retention, which are crucial factors for agricultural production (Huang and Hartemink 2020). Levels of available phosphorus (P) varied substantially among the samples (P1 = 92 mg/dm^3^, P2 = 43 mg/dm^3^, P3 = 29 mg/dm^3^), and all exceeded the recommended minimum threshold for agricultural soils (≥ 20 mg/dm^3^) to support plant growth. Calcium (Ca) levels ranged from 2.00 to 2.50 cmolc/dm^3^, and magnesium (Mg) from 0.75 to 1.20 cmolc/dm^3^, both within the recommended reference ranges. Sodium (Na) levels were low, ranging from 0.02 to 0.06 cmolc/dm^3^, indicating no salinity issues. Potassium (K) concentrations ranged from 0.17 to 0.26 cmolc/dm^3^, also within the recommended reference range according to EMBRAPA (2017). No exchangeable aluminum (Al) was detected in any sample (0.00 cmolc/dm^3^), indicating the absence of aluminum toxicity, which can otherwise inhibit root growth and nutrient uptake (Shetty et al. 2021).

The pH of the soil samples ranged from 6.90 to 7.40, indicating slightly acidic to neutral conditions, conditions that are suitable for most agricultural crops. Degradation of glyphosate increases significantly from pH 3–7, and increase becomes much less noticeable above this pH. As indicated by Chen and Liu (2007) the photodegradation efficiency of glyphosate was found to be 66.9% at pH 2, 36.2% at pH 6 and 49.4% at pH 12. Maintaining a balanced pH is crucial, as it influences the solubility of essential nutrients and the activity of beneficial soil microorganisms (Yadav et al. 2020; Baritz et al. 2021). Although the physicochemical results do not directly indicate the effects of glyphosate, its potential long-term impact on soil health should be considered. Glyphosate is known to chelate cations such as calcium and magnesium, forming complexes that can reduce the bioavailability of these essential nutrients to plants (Bortolheiro and Silva 2021). Glyphosate also strongly binds on soil mineral, thereby impeding the availability of micro- and macronutrients uptake in plants (Mertens et al. 2018). Although low sodium concentrations and the absence of aluminum are positive indicators, the continued use of glyphosate may alter nutrient dynamics over time, thereby potentially compromising soil health (Chávez-Ortiz et al. 2022).

Genotoxicity testing was performed to determine the potential hazard of a chemical or agent for direct or indirect DNA interaction. General toxicity profiles of glyphosate against non-target plant species, microorganisms, lower invertebrates, higher vertebrates and humans were reviewed by Singh et al. (2020). From this review, it could be inferred that glyphosate is less toxic to non-target arthropods, compared to other insecticides such as chlorpyrifos or cypermethrin. As previously demonstrated in the model organism *D. melanogaster* glyphosate exhibits genotoxic and mutagenic properties, increasing risk of genetic damage in this non-target organisms and inducing abnormalities in their life cycles (Galin et al. 2019; Kaya et al. 2000; Muller et al. 2021; Strilbytska et al. 2022). In P1 and P2 cases of our study, treated groups of *D. melanogaster* larvae exposed to WSFs revealed higher DNA damage when compared to control. The dendrogram analysis further highlights Zn and Cr as the elements most highly associated with the genetic damage (DI and DF%).

The present study reinforces the synergistic genotoxic effects of glyphosate and other chemicals. According to Defarge et al. (2018) heavy metals such as arsenic, chromium, cobalt, lead and nickel are detectable in 22 different pesticides, including 11 glyphosate-based formulations, as confirmed by mass spectrometry. When these elements were present in combination, a condition commonly observed in soils, their harmful biological effects are amplified (Shen et al. 2019; Alengebaw et al. 2021; Roddam et al. 2025). As presented by Wang et al. (2020) in a soil study of 16 years long, there was significant accumulation of Zn in soils that received fertilization treatments (chemical, organic and its combination). Due to the heterogeneous nature of the soil matrix, it has not yet been fully elucidated whether the glyphosate is the prior or the only contaminant involved in the toxic effects observed. However, due to the possibility of changing the availability of essential as well as toxic metals that are bound to soil particles, glyphosate might also impact soil life (Mertens et al. 2018). Further research should elucidate the role of glyphosate as a chelator (Mertens et al. 2018), as this is a non-specific property potentially affecting many organisms and processes.

In the microbiological analysis, the genus *Bacillus* was the most prevalent across the collection points, with *Bacillus cereus* being the most frequently identified species, present at eight distinct points. This species is commonly found in diverse environments due to its ability to form resistant spores, which enable survival under adverse environmental conditions, such as high temperatures, UV radiation, dehydration, and exposure to chemical disinfectants and pesticides (Jessberger et al. 2020; Liu et al. 2020; Algammal et al. 2024). Several Gram-negative bacteria were also identified, which play diverse roles in the soil ecosystem. *Enterobacter cloacae*, for example, can be an opportunistic pathogen while aiding in the decomposition of organic matter and nutrient cycling, although its presence may signal fecal contamination. *Stenotrophomonas maltophilia* is known to promote plant growth through the production of auxins and siderophores and exhibits antifungal activity (Ghosh et al. 2020; Hu et al. 2021). *Pseudomonas nitroreducens* contributes to the degradation of organic pollutants and plant growth via production of bioactive compounds (Jayaraj et al. 2023). *Prevotella heparinolytica* is commonly found in the gastrointestinal tract of humans and animals, and its presence in the soil may indicate fecal contamination (Wongkiew et al. 2022). *Rhizobium radiobacter* is recognized for its ability to form nodules on legume roots, promoting nitrogen fixation and plant growth (Atuchin et al. 2023). The presence of these bacteria indicates a rich ecological dynamic, where some species promote soil and plant health, while others may pose contamination risks.

Studies have suggested that glyphosate can exert selective pressure on microbial communities, favoring the proliferation of resistant organism and altering bacterial community composition (Kepler et al. 2020; Raoult et al. 2021; Bearson et al. 2024). The presence of multidrug-resistant *S. maltophilia* at multiple sampling points suggests that this microorganism is particularly adapted to the different contaminants. Consistent with this interpretation, Costa et al. (2022) reported the presence of several bacteria exhibiting resistance to both antibiotics and glyphosate, with multidrug efflux pumps playing a key role in mediating resistance to both agents. In the present study, bacteria identified as resistant to antibiotics and glyphosate include *S. maltophilia*, *K. variicola* and *S. siniae*. Overall, the diversity of bacterial species observed indicates a complex ecological dynamic, in which some species contribute to soil and plant health, while others may result of contamination levels.

## Conclusion

This study has assessed the impacts of glyphosate use in irrigation projects located in the Brazilian semiarid region. While glyphosate remains effective for weed control and enhancing agricultural productivity, the results demonstrate that its application have implications for soil quality and bacterial resistance. The physicochemical analyses of the soil indicated acceptable parameters for agricultural use, although the continuous application of glyphosate may, over time, alter the availability of essential nutrients and soil structural properties. The microbiological assessment revealed substantial bacterial diversity, predominantly within the genus *Bacillus*, alongside the presence of multidrug-resistant bacteria, as *Stenotrophomonas maltophilia*, *Bacillus cereus*, and *Enterobacter cloacae*. Environment genotoxicity analysis conducted with *D. melanogaster* raises concerns in the Icó-Mandantes project area (P1 and P2) due to the possibility that glyphosate persists in the soil, generating toxicity in non-target organisms. Due to the local environmental conditions, characterized by low rainfall (semi-arid environment), low urbanization, and human development based on irrigated agriculture, we assume that glyphosate and its byproducts are present in the soils of the evaluated family farms and are agents that interfere with the quality of these soils. Together, the results reinforce the need for quantification, monitoring of the effects that glyphosate has on soil systems and the adoption of integrated management strategies to reduce dependence on chemical herbicides and limit the proliferation of resistant bacterial strains.

## Electronic Supplementary Material

Below is the link to the electronic supplementary material.

Supplementary Material
