# Supplementary material for: Emerging Bacterial Resistance and Genotoxicity of Water-Soluble Fractions of Agricultural Soils from the Semiarid Region of Brazil Affected by the Continuous Use of Glyphosate
Source: Bull Environ Contam Toxicol. 2026 Apr 6;116(4):85. doi: 10.1007/s00128-026-04230-1 (PMC13053334; doi:10.1007/s00128-026-04230-1)
Supplement: Supplementary file 1 — Supplementary Material [file 128_2026_4230_MOESM1_ESM.pdf]

Supplementary Material

Table S1. Soil density, particle size composition, pH, available phosphorus (P), and exchangeable cations (Ca, Mg, Na, K) were determined following the methodologies described in Manual of Soil Analysis Methods (EMBRAPA 2017).

Soil characterization	Methodology
Soil density (SD)	Soil density (Ds) was determined using the volumetric cylinder method. Undisturbed samples were collected using metal cylinders of known volume, ensuring no compaction during sampling. The samples were then oven-dried at 105°C until a constant weight was achieved. Soil density was calculated as the ratio between the dry soil mass (ms) and the volume of the cylinder (V). The particle density was determined using the volumetric flask method, in which the dry soil samples were transferred into a volumetric flask and filled with ethanol to displace air. The volume of ethanol required to fill the flask was used to calculate the volume of the soil particles.
Particle size composition	Particle size analysis was performed by following the dispersion of soil samples in a sodium hexametaphosphate solution, accompanied by agitation and sedimentation. The fractions of coarse sand, fine sand, silt, and clay were separated through a combination of sieving and sedimentation. Each fraction was dried and weighed, and the results were expressed in g/kg.
pH and available phosphorus (P) determination	Soil pH was measured in a 1:2.5 soil-to-distilled water suspension using a calibrated pH meter. Dry samples were mixed with distilled water, shaken, and allowed to settle prior to measurement. Available phosphorus was determined using the ion exchange resin method. Soil samples were shaken with anion exchange resins, followed by elution of the absorbed phosphorus using a sodium chloride solution. The phosphorus concentration in the extract was determined colorimetrically.
Exchangeable cations	Exchangeable cations (Ca^{2+}-Calcium, Mg^{2+}-Magnesium, K^{+}-Potassium, Na^{+}-Sodium) were extracted using 1 mol/L ammonium acetate solution, pH 7.0. The concentrations of Ca^{2+} and Mg^{2+} were determined by atomic absorption, while K^{+} and Na^{+} were quantified by flame photometry.

Table S2. Bonferroni post-hoc analysis for pairwise comparisons of the mean Damage Index (below the diagonal) and the Damage Frequency (above the diagonal) between the negative control (distilled water), positive control (Cyclophosphamide 2 mg/mL) and water soluble-fraction of soil samples (P1, P2 and P3).

Samples	Negative control	Positive control	P1	P2	P3
Negative control		0.0001*	0.0001*	0.0001*	1.000
Positive control	0.0001*		0.163	1.000	0.0001*
P1	0.0001*	1.000		0.292	0.0001*
P2	0.0001*	0.006*	0.109		0.0001*
P3	0.109	0.003*	0.0001*	0.0001*	

* significant differences ($P \leq 0.05$).

Table S3. Chemical elements (µg/L) detected in the water soluble-fraction of soil samples (P1, P2 and P3).

	Chemical element concentration (µg/L)						
Soil samples	Al	Cd	Co	Cr	Mn	Pb	Zn
P1	53.72<br>(5)	0.008<br>(0.0003)	2.62<br>(0.57)	22.09<br>(6.8)	33.95<br>(0.6)	9.797<br>(2.97)	0.04<br>(0.007)
P2	30.77<br>(3)	0.006<br>(0.0002)	< 0.3	16.79<br>(5.2)	29.97<br>(0.5)	4.765<br>(1.45)	0.03<br>(0.005)
P3	32.62<br>(3)	0.589<br>(0.023)	0.71<br>(0.15)	10.02<br>(3.1)	31.34<br>(0.5)	4.522<br>(1.37)	0.02<br>(0.003)

Values in parentheses refer to the analytical uncertainties expanded at the 95% confidence level.
